# How Action Shapes Body Ownership Momentarily and Throughout the Lifespan

**DOI:** 10.3389/fnhum.2021.697810

**Published:** 2021-07-06

**Authors:** Marvin Liesner, Nina-Alisa Hinz, Wilfried Kunde

**Affiliations:** ^1^Department of Cognitive Psychology, Julius-Maximilians-Universität Würzburg, Würzburg, Germany; ^2^Department of Psychology, Ludwigs-Maximilians-Universität München, Munich, Germany

**Keywords:** body ownership, attentional reweighting, children, haptic neglect, ideomotor theory, ontogeny, perception and action

## Abstract

Objects which a human agent controls by efferent activities (such as real or virtual tools) can be perceived by the agent as belonging to his or her body. This suggests that what an agent counts as “body” is plastic, depending on what she or he controls. Yet there are possible limitations for such momentary plasticity. One of these limitations is that sensations stemming from the body (e.g., proprioception) and sensations stemming from objects outside the body (e.g., vision) are not integrated if they do not sufficiently “match”. What “matches” and what does not is conceivably determined by long–term experience with the perceptual changes that body movements typically produce. Children have accumulated less sensorimotor experience than adults have. Consequently, they express higher flexibility to integrate body-internal and body-external signals, independent of their “match” as suggested by rubber hand illusion studies. However, children’s motor performance in tool use is more affected by mismatching body-internal and body-external action effects than that of adults, possibly because of less developed means to overcome such mismatches. We review research on perception-action interactions, multisensory integration, and developmental psychology to build bridges between these research fields. By doing so, we account for the flexibility of the sense of body ownership for actively controlled events and its development through ontogeny. This gives us the opportunity to validate the suggested mechanisms for generating ownership by investigating their effects in still developing and incomplete stages in children. We suggest testable predictions for future studies investigating both body ownership and motor skills throughout the lifespan.

## Introduction

What counts as a person’s body? When looking at other living agents, most of them appear to have a more or less clearly circumscribed body, which is separated from other objects and other agents. Thus, the body of other agents is an object that can be distinguished from other objects by all the perceptual means that apply to separating objects from each other (such as figure-ground segmentation and gestalt factors of perception).

Yet, when agents perceive their own body, the matter of affairs seems to become more complicated. Of course, an agent’s body is a distinct object, like all other objects, and can thus be separated from other objects by the same means as mentioned before. But what makes it unique? How is the biological body experienced as not just another object in the environment, but as being “owned” by oneself? The crucial factors seem to relate to interoception^1^, which can be passively experienced or actively generated, as discussed in the following.

### “Passive” Coincide of Interoceptive and Exteroceptive Signals

An agent’s body provides sensory signals that are accessible to only the agent herself. These are interoceptive signals, which result in tactile, proprioceptive, and kinesthetic perception. Thus, interoceptive signals are unique in the sense that only one object in the perceptual world generates such signals, namely the object that is called own “body”, whereas other objects do not. For example, agents can see that two objects touch each other so as they can see that an object touches the own body. Yet, only the own body generates the experience of touch. Interoceptive signals thus provide a very strong and unambiguous cue of ownership. The special role of interoceptive signals is also underlined by the existence of neuronal pathways and brain regions like the insular, anterior cingulate, or somatosensory cortex which are specialized in processing these interoceptive signals (Critchley et al., 2004; Craig, 2009).

However, an organism can perceive exteroceptive signals as well, i.e., signals that originate from locations other than that of the sensors which encode them (e.g., light reflected by an object creating a visual sensation) and also for these specific neuronal pathways exist (e.g., visual cortex: Grill-Spector and Malach, 2004; auditory cortex: Romani et al., 1982; Belin et al., 2000). Obviously, we see parts of our body (such as our hands) quite often, and other agents can also see them. If a body limb is touched, the agent feels and sees that touch so both interoceptive and exteroceptive perceptual information is available. Interestingly, visual changes that are accompanied by corresponding tactile changes are judged as belonging to the agent herself. This is the basic idea behind the rubber hand illusion and its various versions (e.g., Botvinick and Cohen, 1998; Armel and Ramachandran, 2003; Sanchez-Vives et al., 2010; Kalckert and Ehrsson, 2012; Ma and Hommel, 2015a, b; Cardinali et al., 2021). In the original experiment by Botvinick and Cohen (1998), participants received brush strokes on their occluded hand while simultaneously watching a fake hand in front of them being stroked synchronously or asynchronously with their real hand. While participants had the illusory experience that the artificial hand was part of their own body in the synchronous condition, this was not the case or to a much lesser extent in the asynchronous condition. That the system ascribes ownership to such artificial objects like fake hands comes across in different ways. First, people report experiencing ownership when being asked (e.g., Dummer et al., 2009; Rohde et al., 2011; Kalckert and Ehrsson, 2012; Ma and Hommel, 2013, 2015b; Liesner et al., 2020a). Second, the felt position of a touched body part moves towards the object that is seen to be touched (proprioceptive drift; e.g., Dummer et al., 2009; Rohde et al., 2011; Kalckert and Ehrsson, 2012; Liesner et al., 2020b). Third, there are neural (Ehrsson et al., 2004; Makin et al., 2008) and several physiological responses to these manipulations such as a temperature decrease of the stimulated body part (e.g., Moseley et al., 2008; Hohwy and Paton, 2010; van Stralen et al., 2014) and increased skin conductance responses when the observed external object is threatened (e.g., Armel and Ramachandran, 2003; Ma and Hommel, 2013, 2015a).

Following this ground-breaking observation it has been suggested that the human perceptual system is biased towards ascribing body ownership to essentially any object that generates exteroceptive signals (e.g., Gallagher, 2000; Verschoor and Hommel, 2017), providing they sufficiently coincide in a spatial-temporal manner with interoceptive signals (e.g., Botvinick and Cohen, 1998; Suzuki et al., 2013; Kalckert and Ehrsson, 2014; Tajadura-Jiménez and Tsakiris, 2014; Ma and Hommel, 2015a, b). This relatively “unselective” approach has however been criticized recently, the reasons for which we will discuss throughout this article.

### Constraints of Passive Ownership and Developmental Factors

The experience of ownership in passive agents is constrained in various ways. As said before a sufficient spatial and temporal coincidence of exteroceptive and interoceptive signals is necessary. In fact, the comparison between synchronous and asynchronous stimulation has become a kind of gold standard to indicate ownership experience (Botvinick and Cohen, 1998; Ehrsson et al., 2004; Tsakiris and Haggard, 2005; for a critical assessment see Kalckert et al., 2019a).

Physical resemblance (i.e., “corporeality”) of the seen body part to the own body seems important as well. By and large, the less similar an object is to an agent’s body parts, the lower is experienced ownership (Tsakiris et al., 2010; Guterstam et al., 2013). Here it is important to critically evaluate the synchronous-asynchronous index mentioned before. For example, it might well be that people report more experienced ownership in the former than latter condition with all kinds of objects they see. Yet, the absolute level of the body ownership experience with non-corporeal objects is often way below what people report with a body-similar rubber hand (e.g., Kalckert et al., 2019a) and it is unclear from which level of reported experience on an “authentic” feeling of bodiliness should be assumed (see Liesner et al., 2020b, for similar arguments). Therefore, besides investigating difference scores, researchers should carefully take into account the absolute level of ownership measures and critically evaluate which conclusions can and cannot be drawn from their measures. This especially applies to explicit ratings of ownership which might be prone to demand effects (Orne, 1962) since participants might feel “committed” to respond differently to different manipulations. Additionally, for highly corporeal objects some level of ownership experience has been reported even in the absence of any stroking, though lower compared to conditions with stroking, suggesting that the experience is elicited easily with these objects (Rohde et al., 2011; Samad et al., 2015). Despite quantitative changes, even the qualitative aspect of ownership might change with more or less corporeal objects which are stroked or actively controlled. For example, while even in the most realistic settings using the rubber hand illusion, most participants still explicitly “know” that the seen rubber hand is not actually part of their body, they nevertheless have the experience that they feel the brush stroke on the rubber hand and report that it feels like it would be their own hand (e.g., Botvinick and Cohen, 1998). However, while even with non-corporeal objects (e.g., wooden blocks, balloons, cursors), several measures (e.g., proprioceptive drift, skin conductance response) might still suggest the presence of the illusion, it is much less likely that participants report to “feel” the stroke on the artificial object, let alone rationally accept it as part of their own body (e.g., Ma and Hommel, 2015a; Kalckert et al., 2019a; Liesner et al., 2020b). In fact, it has been suggested by most studies that illusory (explicit) ownership cannot be experienced for non-corporeal objects at all (e.g., Tsakiris and Haggard, 2005; Tsakiris et al., 2010; Guterstam et al., 2013; Kalckert et al., 2019a). There are a few noticeable studies from recent years which question this constraint which we will discuss in the section “Does Active Ownership Depend on Immediate Control Experience?” (e.g., Liepelt et al., 2017; Cardinali et al., 2021). Full-body illusions (e.g., Slater et al., 2010), a paradigm in which a complete virtual body is looked at by the participant which either receives (a)synchronous tactile stimulation with the participant or is (a)synchronously controlled by the participant, might provide the opposite end of a corporeal-to-non-corporeal continuum. While grounding on the same mechanisms of visuotactile or visuomotor matching as the rubber hand illusion, it has been shown that the more realistic a virtual body looks, the less additional multisensory stimulation is needed to induce an ownership illusion (Maselli and Slater, 2013; Kilteni et al., 2015; O’Kane and Ehrsson, 2021).

In the same vein, it seems as if a biologically plausible position of the external object is necessary (e.g., Ehrsson et al., 2004) to experience ownership over it. Ownership over the (stroked) body-external object decreases with increasing distance to the participant’s real limb until it vanishes completely (e.g., Lloyd, 2007; Kalckert et al., 2019b).

From a developmental perspective, it would be interesting to study whether the requirement of visual and/or anatomical resemblance is a matter of own visual experience of body parts, or perhaps more or less innate. This question is particularly pertinent for certain versions of ownership “illusions” like the impression that a seen face is the own face when being concurrently touched (enfacement illusion; e.g., Tajadura-Jiménez et al., 2012). The visual experience of the own face is, in any case, limited to technically supported instances like mirrors. Additionally, at a young age where such instances have not occurred that often, children might be even more limited regarding a visual representation of their own face. In line with this, Brownell et al. (2010) demonstrated that children below 2.5 years of age have considerable problems in identifying their own corresponding body parts when asked to match them with the body parts of an observed person which the authors interpreted as evidence for a less developed representation of the own body in these children. Consequently, the limited visual experience with their own face in young children might facilitate the “embodiment” of other faces, as there is not yet a visual standard that runs counter to this perceptual interpretation (Filippetti and Tsakiris, 2018). It has been suggested that such a standard for a “robust” face representation is only acquired by extensive visual experience with a face and that even highly familiar faces might still gradually differ from each other regarding the robustness of such a representation (Tong and Nakayama, 1999; Caharel et al., 2002). Multiple studies have shown that the right temporoparietal junction is of high relevance for recognizing one’s own face providing a possible neural basis for such a robust face representation (e.g., Decety and Lamm, 2007; Heinisch et al., 2011; Zeugin et al., 2020). However, while these findings and arguments suggest that the limited visual experience with one’s own face might facilitate ownership experiences in the enfacement illusion, this should only lead to gradual differences in ownership experiences so that the basic mechanisms discussed in this review which are mainly based on observations from the rubber hand illusion should also account for other body ownership illusions like the enfacement or full-body illusion (e.g., Slater et al., 2010; Maselli and Slater, 2013).

Likewise, it is important to study whether the requirement of “coincidence” of interoceptive and exteroceptive signals is innate or a matter of experience. In other words, must an agent have experienced that a certain touch typically goes along with a visually accessible object to ascribe ownership to that visual object? Some interesting insights on this might be taken from studies investigating mirror-self-recognition which suggest that, especially at an early age, immediate current visuomotor matching might play an important role in the ability to pass self-recognition tests such as the mark task (e.g., Merleau-Ponty, 1982; Mitchell, 1993). It has however been criticized that these studies might not represent "actual" understanding of the visual representation of oneself in the mirror. Instead, young children might just be highly sensitive for visuomotor synchronies and therefore simply notice the matching contingencies between sensorimotor and visual perceptions when moving in front of a mirror, without necessarily “understanding” that they see themselves in the mirror (Mitchell, 1993). The notion of children’s high sensitivity for visuomotor synchronies is also supported by various studies demonstrating that children already show differentiation between synchronously and asynchronously presented visual and tactile stimulation within the first year of life (Bahrick and Watson, 1985; Zmyj et al., 2011; Filippetti et al., 2013, 2015 though see Maister et al., 2020 for possible limitations to this). This should then also account for the child’s own body, which is supported by a study from Bigelow (1981) demonstrating that children in their second year of life recognize themselves earlier in conditions in which synchronous movement feedback is provided (e.g., a mirror) than in conditions without movements (e.g., a photograph). Supporting our previous suggestion that embodiment of external objects might be very flexible in children and that this flexibility should decrease with accumulating knowledge about “typical” multisensory or sensorimotor experiences, it has also been shown that the rubber hand illusion effect in children as compared to adults, is larger (Cowie et al., 2016) or less constrained to synchronous conditions or to the application of stroking at all (Cowie et al., 2013; Nava et al., 2017). This high sensitivity for visuomotor matching and flexible and less restrained inference of ownership might be extremely important for children in order to learn a consistent body model through actively generating sensory signals.

There is however also a very different way of interpreting the previously mentioned findings on children’s sensitivity for synchronous and asynchronous visual and tactile stimulation (Bahrick and Watson, 1985; Zmyj et al., 2011; Filippetti et al., 2013, 2015). Tsakiris (2010) suggested a multi-step model of body-ownership in which incoming sensory information is first tested against a fixed body model before a potential multisensory contingency is detected and a sense of (body) ownership is inferred. This assumes a more or less innate body model independent from the learning experience, whose neural basis might be located in the temporoparietal junction (Tsakiris et al., 2008). According to Tsakiris (2010), the findings that (passive) ownership often cannot be elicited with non-corporeal objects or objects in an anatomically implausible position provide evidence for such a fixed body model (Ehrsson et al., 2004; Tsakiris and Haggard, 2005; Lloyd, 2007; Tsakiris et al., 2010; Guterstam et al., 2013; Kalckert et al., 2019a, b). Besides, Morgan and Rochat (1997) observed that already 3-month olds could distinguish between mirrored and unmirrored real-time videos of their own moving legs. However, even at 3 months of age children have already gained considerable sensorimotor experience, and also other (ir)regularities limiting ownership might just as well be learned based on experience. Nevertheless, the two accounts might not be that incommensurable after all since even fetuses presumably already collect some sensorimotor experience *in utero* so that an innate body model might be based on such prenatal experiences as well.

### “Active” Generation of Coinciding Interoceptive and Exteroceptive Signals and “Active Ownership”

Ownership can also originate from an agent’s efferent activity (e.g., Sanchez-Vives et al., 2010; Kalckert and Ehrsson, 2012). In these cases, the agent creates the sort of interoceptive-exteroceptive coincidence that generates ownership experience herself. For example, if hand muscles are contracted this comes with proprioceptive and visual experiences at the same time. As with passive stimulation discussed in the preceding paragraph, ownership by self-stimulation goes beyond objects that resemble typical body parts. Everyday experience and scientific studies suggest that all kinds of objects an agent actively controls by body movements, such as tools (e.g., Maravita et al., 2002; Weser et al., 2017), sports gadgets or virtual objects (e.g., Ma and Hommel, 2015a, b; Kirsch et al., 2016; Liesner et al., 2020a, b) can be experienced by the agent as belonging to her body to some degree as indicated by neural, physiological, explicit and implicit behavioral measures.

Despite the coincidence of interoceptive and exteroceptive signals, a very important factor shaping “active” ownership experience is that perceptual changes that occur after efferent activity were predicted or anticipated prior to these efferent activities. In other words, the perceptual changes caused by motor activity must be controllable, to create an experience of agency (Haggard, 2017). This sort of active ownership experience can thus be called “ownership by agency”. In fact, it has been proposed that the controllability of perceptual events is the key, if not the only, factor for ascribing ownership to these events (Verschoor and Hommel, 2017, “self by doing approach”). In a nutshell, this approach claims that every perceptual change that is foreseeably caused by efferent activity counts as body suggesting a bottom-up approach of ownership where perceptual input is simply integrated with any motor activity producing it (e.g., Botvinick and Cohen, 1998; Armel and Ramachandran, 2003; Ma and Hommel, 2015a). This is a very optimistic approach regarding the extension of ownership to external events since it does not only suggest that a sense of ownership is triggered by control experience over perceptual changes, but also that any such control experience should lead to the ascription of ownership to the manipulated object. If it was correct, there was neither room for a distinction between the sense of agency and sense of ownership nor for a special role of interoceptive effects of motor activities for generating ownership experience, provided exteroceptive effects are sufficiently predictable. Agents with absence or loss of interoception provide an interesting testbed for this proposal (e.g., Gallagher and Cole, 1995). Furthermore, according to this reasoning, an agent should also not be able anymore to distinguish between different components of an action like a body effector, a tool, or an object in the environment that is acted upon. For example, when using a hammer to put a nail into a wall, perceptual input from the hand (proprioceptive, tactile), the hammer (visual), and the nail (visual) are all equally predictable and controllable, but does this mean that they are also ascribed ownership equally? We believe that this is too far-fetched since again, differences between interoception and exteroception need to be accounted for.

### Constraints of Active Ownership

One constraining factor regarding active ownership is the anatomical resemblance. As with passive stimulation methods, most studies investigating ownership for corporeal objects showed larger illusion effects as compared to non-corporeal objects (e.g., Tsakiris and Haggard, 2005; Tsakiris et al., 2010; Guterstam et al., 2013; Kalckert et al., 2019a). However, this difference seems to be smaller for active ownership than for passive ownership (Ma and Hommel, 2015b; Liepelt et al., 2017; Zopf et al., 2018). While a sense of ownership is very limited for non-corporeal objects with passive stimulation (Tsakiris and Haggard, 2005; Tsakiris et al., 2010; Guterstam et al., 2013; Kalckert et al., 2019a), it might still be possible with active movements. However, this seems to be restricted to implicit measures such as proprioceptive drift (Liesner et al., 2020b). Additionally, similar to passive ownership illusions, active ownership illusions have been shown to be disrupted by temporal asynchrony between interoceptive and exteroceptive sensations (Dummer et al., 2009; Kalckert and Ehrsson, 2012, 2014; Ma and Hommel, 2013, 2015a,b) and increasing distances between the biological body and the body-external object (Liesner et al., 2020b).

There is however another important constraint in active ownership, which relates to the processes of generating efferent activities in the first place. While actively operated tools can be ascribed ownership, this does not occur, if the spatial discrepancy between felt and seen movements exceeds a certain level, despite identical levels of (complete) predictability (Liesner et al., 2020a,b). In the studies by Liesner et al. (2020a, b); participants moved a cursor on a computer screen by spatially compatible or incompatible hand movements, i.e., by hand movements in the same or opposite direction. Subjective ownership ratings were higher in the compatible than in the incompatible condition, and only with compatible tool movements was proprioceptive drift significantly different from a non-control baseline condition (Liesner et al., 2020b). Interestingly, the sense of agency seems to be affected similarly by such discrepancies between interoceptive and exteroceptive signals (e.g., Ebert and Wegner, 2010; Liesner et al., 2020a), supporting the idea that the experience of agency and ownership are correlated in these situations and that the sense of agency could be a factor underlying the experience of active ownership. But why should spatial discrepancy have such detrimental effects on the sense of agency and ownership despite an identical objective level of predictability? There is ample evidence that human agents generate motor activities by recollecting the perceptual changes these motor activities produce according to previous experience (e.g., Elsner and Hommel, 2001; Kunde, 2001; Liesner et al., 2020a). This is the so-called *ideomotor approach* to action control (e.g., Koch et al., 2004; Shin et al., 2010; Waszak et al., 2012; Hommel, 2013). In the case of incompatible hand and object movements, the anticipated perceptual changes are interfering because the anticipated inverted movements of hand and object contradict the common experience that objects controlled by one’s hand should move in the same direction as the hand. This interference, which is already present at movement planning, thus seems to disrupt the integration of interoceptive and exteroceptive sensations in terms of ownership experience as well.

Human agents amass a lot of experience with the interoceptive (e.g., proprioceptive, kinesthetic) effects of their motor activities, except in rare cases of loss of body-related perception which will be discussed later (section “Development of Active Ownership”). James (1981) called these effects “resident” as they almost insurmountably accompany bodily movements and thus “reside” within or on the body. So in neurotypical agents, interoceptive signals are not only unique in the sense, that just one object in the world can generate that experience. They are also unique in the sense, that they are very closely linked to the agent’s efferent activities, conceivably much closer than any other possible exteroceptive effect of motor activities, both, in terms of spatial proximity and ubiquity. As explained above, efferent activities mostly produce interoceptive as well as exteroceptive effects, and agents can access motor patterns based on both. Thus, we can feel and see a hand moving and can generate that movement by imaging the visual or proprioceptive effects of doing so (Pfister, 2019). Which of these effect codes are eventually engaged is a matter of instruction (Memelink and Hommel, 2013; Mocke et al., 2020). It is also a matter of the compatibility between interoceptive and exteroceptive effects. If agents aim at certain exteroceptive effects which, however, go along with spatially incompatible interoceptive effects, such as when operating tools that move in directions opposite to the operating hand, this typically comes with performance costs (Kunde, 2001; Kunde et al., 2007; Müsseler and Skottke, 2011; Kunde et al., 2012; Wirth et al., 2015; Liesner et al., 2020a, b). Agents aim to overcome such performance costs by downregulating the less task-relevant effect component during action generation (Fourneret and Jeannerod, 1998; Knoblich and Kircher, 2004; Sülzenbrück and Heuer, 2009; Liesner and Kunde, 2020; Liesner et al., 2020b) which in tool use are interoceptive representations. This downregulation of interoceptive codes in tool use has been named “haptic neglect” (Heuer and Rapp, 2012) and can be understood as an attentional shift away from sensory signals emerging from the body and towards sensations emerging from the controlled tool. It is tempting to assume that it is exactly this downregulation of interoceptive codes in situations of discrepant interoceptive and exteroceptive action effects that prevents the integration of temporally contingent visual and interoceptive signals from the same action, which is key to ascribe ownership to visual objects (Gallagher, 2000; Tsakiris, 2017, see section “Linking Action Control and Active Ownership”). This idea has to be tested empirically though.

### Does Active Ownership Depend on Immediate Control Experience?

The previously discussed studies have revealed the pivotal role of active control for the illusion of ownership for non-corporeal objects. It is however unclear whether the experience of ownership for these objects is limited to the narrow temporal windows in which this active control is experienced or outlasts the duration of immediate control over the object. In a recent study, Pfister et al. (2020) investigated this topic in an active rubber hand illusion in which they linked the tapping of participants’ index fingers to the movements of a rubber hand. After 2 min of tapping, participants were asked to stop and simply look at the rubber hand for another 2 min. The authors collected subjective ownership ratings for the rubber hand both after the 2 min of tapping and the 2 min of looking at the rubber hand. Subjective ownership significantly decreased in the 2 min after participants had stopped tapping, but even after the 2 min of inactive observation, subjective ratings were still relatively high (around 5 on a 0–10 scale). Taking a cautious interpretation of absolute values of ownership into account, this study provides the first evidence that even after discontinuation of matching interoceptive and exteroceptive sensations, ownership can be experienced to some (reduced) degree. This suggests that not only present but also past agency experience with an object can shape ownership experience. In a more radical approach, Liepelt et al. (2017) used the passive rubber hand illusion paradigm and compared conditions in which the rubber hand was stroked with conditions in which the participants’ cell phones or a computer mouse were stroked (a)synchronously with the participants’ hands to investigate how the past experience of agency with these objects shapes possible ownership experience in the absence of immediate, current control experience and thus in the absence of immediate sensorimotor matching between interoception and exteroception. The authors found significant differences between synchronous and asynchronous stroking conditions for all tested objects, both regarding subjective ownership ratings and regarding proprioceptive drift, even though these effects were larger for the rubber hand than for the mouse and cell phone. Interestingly, the effects for the latter objects were however larger than for an additionally used wooden block (Liepelt et al., 2017, Experiment 2), an object with supposedly no experience of control over. These results suggest that in addition to concrete and recent sensory matching of interoceptive and exteroceptive signals when controlling an external object, also more complex and long–term experience of action control over external (non-corporeal) objects can lead to the feeling of ownership over these objects.

A related open question is whether mere knowledge of controllability of an object is sufficient to experience ownership over this object independently of any direct control experience. Such situations can, for example, occur with different kinds of tools which people basically know how to use but have not done so before. Cardinali et al. (2021) tested this idea by also adapting the passive rubber hand illusion, but this time using a mechanical gripper instead of a rubber hand and a balloon as a control object. While neither object resembles body parts, the illusion was elicited in terms of proprioceptive drift, subjective ratings, and skin conductance response for the gripper but not the balloon (Experiments 1 and 3) even without previous use of the gripper (Experiment 2). These results suggest that mere knowledge of sensory correlations between the body and object movements can trigger ownership experiences for external objects, possibly by means of activated action plans (see Kirsch and Kunde, 2019).

Knowledge of tool use can originate from observation (e.g., Want and Harris, 2002; Flynn, 2008; Paulus et al., 2011). This is usually explained by the observer forming associations between the observed tool changes and the actions of the observed person triggering these changes (Paulus, 2012, 2014). Thus, by observing other people’s actions, humans can learn the correlations between exteroceptive and expected interoceptive sensory effects of these actions. While we are currently not aware of any studies investigating whether knowledge about the controllability of objects gained from such observation can support the feeling of ownership when later confronted with the object oneself, this might certainly be an interesting question for future research.

A further open question regarding the influence of movements on ownership illusions is whether active control and agency or a pure match between interoceptive and exteroceptive signals that come with actively moving is the driving factor behind the ownership experiences. A way to disentangle these possible influences might be to investigate the impact of passively moving a bodily effector which triggers movement effects in an artificial object. Participants undergoing such an approach would essentially lack the processes of planning and generating these movements themselves and presumably also the experience of agency since they would not actually be “controlling” the external effector in this situation. In the rubber hand illusion, it has been demonstrated that active control is necessary for the sense of agency, but not for the sense of ownership (Kalckert and Ehrsson, 2012, 2014). Kalckert and Ehrsson (2012, 2014) compared the effects of actively controlling a rubber hand and the effects of passive “control” over the rubber hand (the experimenter moved the participant’s real hand and the rubber hand). While ownership over the rubber hand was elicited in both conditions (although smaller in the passive condition), a sense of agency only resulted in the active condition. It is not clear, however, how these findings would translate to non-corporeal objects such as tools and how they would interact with the other factors we have discussed. Especially the situation of incompatible interoceptive and exteroceptive perceptions would be interesting to study in this context since there could be no interference stemming from movement planning anymore. Research on *sensory attenuation* suggests that events are perceived differently when they are effects of one’s own action compared to when the same events are presented without such a previous action (e.g., Voss et al., 2008; Desantis et al., 2012; Brown et al., 2013; Hughes et al., 2013) which is why it is often also used as a measure for a sense of agency (e.g., Braun et al., 2018). However, it is up until now debated whether this effect is based on mechanisms related to the action itself or rather to more general prediction processes (e.g., Kaiser and Schütz-Bosbach, 2018; Klaffehn et al., 2019). Self-induction of interoceptive and exteroceptive changes might not be necessary for a sense of ownership, providing that the input is sufficiently predictable. Interestingly, temporal binding which is often regarded as an implicit measure for the sense of agency (*intentional binding* e.g., Haggard, 2017), does not differ between active or passive finger movements if appropriate control conditions are considered (Kirsch et al., 2019). So measures of the sense of ownership might produce similar results.

Finally, past control experience may play a role in the formation and maintenance of a sense of ownership in clinical cases of paralysis caused by, for example, spinal cord injury. In these patients, afferent and efferent signals cannot be processed beyond the location of the injury which almost always leads to a loss of the ability to generate motor actions and often also limited processing of sensory input from the affected body parts (Lenggenhager et al., 2012; Lucci and Pazzaglia, 2015). However, processing of these signals had been intact in many of these patients for a long time before the incidence, posing the question of how these past experiences can still shape the sense of ownership of the affected limbs. Pozeg et al. (2017) compared paraplegic patients and healthy controls when applying a passive full-body illusion and a passive virtual leg illusion. While they found no group differences in the full-body illusion, experienced ownership for the virtual leg was significantly lower in patients than in controls. Moreover, ownership measures in patients were negatively correlated with the time since the onset of the condition. Even though the illusion in this study was induced by passive stimulation, these results suggest that the “possibility” to act, and previous sensorimotor experience support the formation of the sense of ownership, specifically for the affected limb. Additionally, attempts to re-establish sensorimotor functions in spinal cord injury patients by physiotherapy or passive motor stimulation have beneficial effects on other body-related cognitive processes such as the processing of peripersonal space or body positions (e.g., Scandola et al., 2019, 2020). Given the overlap between these processes and the sense of body ownership, it would be interesting to see whether body ownership could also be strengthened by applying such external motor stimulation similar to active control experience. Besides motor restrictions, spinal cord injuries often come with painful experiences from the paralyzed body parts (van Gorp et al., 2015). Interestingly, some of these painful sensations have also been shown to be reduced by the application of ownership illusion methods to the affected limbs, possibly because the experience of ownership over an artificial limb decreases sensory processing in one’s own limb (Pazzaglia et al., 2016; Pozeg et al., 2017). Therefore it might be promising to integrate methods to induce external ownership, possibly by reactivating previous experiences of control or applying external motor stimulations in therapy and rehabilitation programs to help restoring normal levels of body ownership, body-representation, and body-related sensations in these patients.

### Active Ownership and the Sense of Agency

Some studies have shown high correlations in explicit agency and ownership ratings in active object control which has led some researchers to equalize these two concepts (e.g., Ma and Hommel, 2015a, b). This reasoning is however in contrast with studies suggesting a differentiation between the sense of agency and the sense of ownership (e.g., Gallagher, 2000; Jeannerod, 2003; Tsakiris et al., 2007; Kalckert and Ehrsson, 2012). Moreover, ownership of a rubber hand can occur regardless of whether touch is actively generated or passively imposed (Tsakiris et al., 2006; Riemer et al., 2013) while it might be more expressed with active generation (Dummer et al., 2009; Kokkinara and Slater, 2014). These observations with rubber hands are in stark contrast to the findings reviewed above which suggest the necessity of active control for the emergence of ownership experiences with non-corporeal objects (Maravita et al., 2002; Ma and Hommel, 2015a, b; Kirsch et al., 2016; Weser et al., 2017; Liesner et al., 2020a, b). Thus, while for objects with visual similarity to the biological body like rubber hands, active control over these objects might play a smaller role in constructing a sense of ownership, for less corporeal objects, actual (or remembered) control seems key. Conceivably, there is a higher chance to integrate objects into one’s body which also resemble the body compared to non-corporeal objects. For non-corporeal objects like cursors or geometrical objects, however, the initial likelihood that these are regarded as part of one’s body might be generally very low so that additional control experience from visuomotor matching might have a stronger impact on the sense of ownership for the external object. Similarly, also in the full-body illusion, it has been shown that the more realistic a virtual body looks, the less important becomes additional control over the virtual body for an ownership illusion to emerge (Slater et al., 2010; Maselli and Slater, 2013). All these observations neatly fit with the sense of ownership constructed as a Bayesian information integration approach as suggested by Samad et al. (2015).

### Linking Action Control and Active Ownership

**Figure 1:**
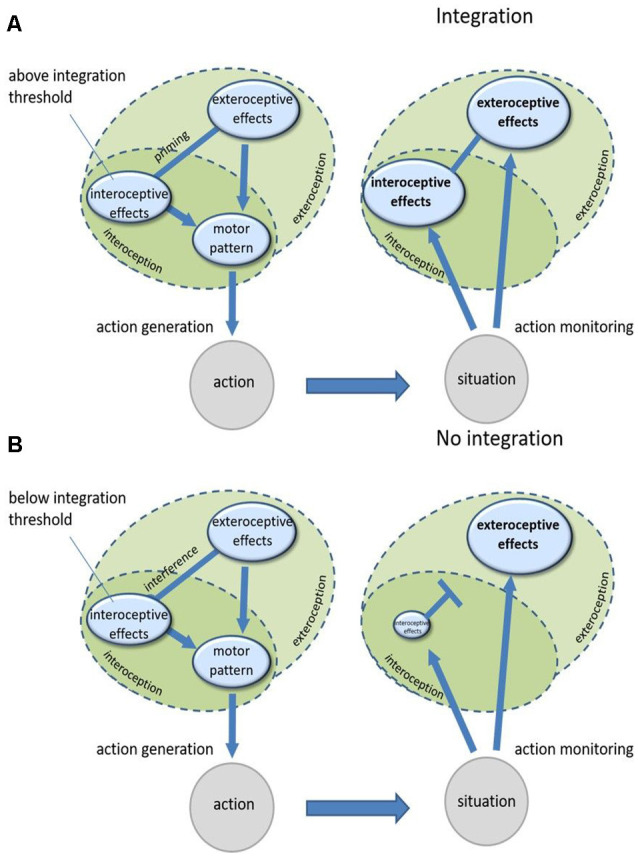
**(A)** Controlling an object that produces exteroceptive effects which are compatible with the accompanying interoceptive effects. Both effect representations are linked to the same motor patterns and therefore prime each other when generating the action so that both are highly activated. When monitoring the effects of the action, both their representations are highly activated which enables their integration. **(B)** Controlling an object that produces exteroceptive effects which are incompatible with the accompanying interoceptive effects. Both effect representations are linked to interfering motor patterns so that one of them (mostly interoceptive effects) needs to be suppressed for action generation. When monitoring the effects of the action, interoceptive effects are then suppressed to a degree where they cannot be integrated with exteroceptive effects anymore.

Action planning essentially depends on previous experience with the action and the effects which are usually produced by it. Performing an action creates bidirectional links between motor codes of this action and its associated typical sensory effects, both interoceptive and exteroceptive ones (Koch et al., 2004). As explained above, integration of an actively controlled body-external object with one’s body is countermanded by the interference of exteroceptive information (from the object) and interoceptive information (from the body) that contradicts the previously learned links between an action and its effects (Ebert and Wegner, 2010; Liesner et al., 2020a, b). In cases of such interference, agents tend to downregulate one of the two components, mostly the interoceptive component, in a seemingly strategical top-down process (Fourneret and Jeannerod, 1998; Knoblich and Kircher, 2004; Müsseler and Sutter, 2009; Sülzenbrück and Heuer, 2009; Heuer and Rapp, 2012; Liesner and Kunde, 2020; Liesner et al., 2020b). This downregulation probably facilitates the generation of actions with interfering interoceptive and exteroceptive information but impairs the integration of actual interoceptive and exteroceptive signals once they occur during movement execution. In a nutshell, to initiate an action, agents seek to overcome the interference of interoceptive and exteroceptive signals. They do so by downregulating, or “attending away” from, the interoceptive effect component. This interference-caused downregulation before action onset subsequently continues during movement execution and hinders the integration of actual interoceptive and exteroceptive signals after action onset because of the low representational strength of the interoceptive signals (see Figure 1B for an illustration). In the case where interoceptive and exteroceptive information are compatible and thus do not interfere during an action, there is no need for such downregulation since both anticipated interoceptive and exteroceptive effects can be used for action generation. Without downregulation, actual interoceptive and exteroceptive effects can be integrated into these situations easily (Figure 1A). While this model is mainly designed to explain differences in ownership experiences with immediate control experiences, it can also account for the findings discussed previously that past control experience alone can in some cases elicit ownership experiences as well (Liepelt et al., 2017; Cardinali et al., 2021; Pfister et al., 2020). When presenting an object with which a high amount of control experience has been accumulated in the past, the interoceptive and exteroceptive effect codes associated with controlling this object might already be activated to a degree which leads to their integration without a need to perform the action.

Furthermore, additional evidence for the high interrelatedness of action control mechanisms and the experience of ownership stems from studies investigating the neural correlates of both these processes. For example, Evans and Blanke (2013) observed similar mu activity in sensorimotor, premotor, and posterior parietal cortices when participants were experiencing a virtual hand illusion and when they were engaging in a motor imagery task. These results are mirrored by various studies which have demonstrated neural activity in these areas both during ownership illusions (e.g., Ehrsson et al., 2004; Makin et al., 2008) and when engaging in motor planning or motor execution (e.g., Overney and Blanke, 2009; Ionta et al., 2010). Interestingly, Perruchoud et al. (2016) demonstrated that these areas showed specific activation patterns when participants performed a mental rotation task with pictures of hands, but not with pictures of full bodies. While the former task might put a stronger emphasis on sensorimotor simulation the latter might be more related to mental frame of reference rotations.

That downregulating of interfering interoceptive sensations can benefit action control is suggested by the performance of “deafferented” patients, i.e., patients with intact efferent pathways but a more or less complete loss of interoception (e.g., Taub, 1976; Cole and Paillard, 1995). Interestingly, deafferented patients do not show the performance drop in “mirror drawing”, where one only sees the mirror image of one’s drawing hand while copying an image, compared to standard drawing conditions that neurotypical humans normally show (Lajoie et al., 1992). When neurotypical agents mirror-draw there is a mismatch between visual and proprioceptive information, which obviously cannot occur in patients that lack the proprioceptive component. While such “forced haptic neglect” seemingly helps to perform goal-directed movements in situations which usually pose difficulties for action generation, it also has a strong impact on the way these patients perceive their own body and self (Cole and Paillard, 1995; Gallagher and Cole, 1995; Renault et al., 2018). For example, Cole and Paillard (1995) report that one of the “deafferented” patients experienced a “floating” feeling without any sense of body ownership in the first time after the onset of his condition while another patient often refers to her body as an external “tool” or “machine” rather than something which is part of herself. Interestingly, highly similar subjective experiences of a feeling of “losing” one’s body have been reported by users of psychedelic drugs which disrupt proprioceptive sensations (Millière et al., 2018).

The loss of the sense of (body) ownership in cases of deafferentiation fits well with the observation that neurotypical agents experience less, or even no, sense of ownership over controlled objects in situations where agents downregulate interoception in service of action control. Such downregulation might in fact be construed a temporary “deafferentiation”. The question however remains on which basis a system “decides” that incoming sensory information from interoception and exteroception interferes to a degree so that downregulation becomes necessary, which then limits the potential to experience ownership.

### Development of Active Ownership

Ideomotor theory suggests that agents generate motor activities by the recollection of the perceptual effects of these motor activities which then, in turn, activates these motor activities (e.g., Koch et al., 2004; Shin et al., 2010; Waszak et al., 2012; Hommel, 2013). This however requires that the agent must have accumulated a sufficient amount of experience regarding which motor activities produce which perceptual sensations. It is assumed that this happens based on “motor babbling” in children, i.e., explorative “random” movements through which the child builds associations between specific movements and their sensory effects (e.g., Paulus et al., 2012). Based on this conjecture, also interference between interoceptive and exteroceptive sensations is based on the experience of common action-effect links, or, more specifically, on their violation. Indeed, what is “interfering” in situations in which we commonly observe, for example, difficulties in action generation, is the combination of current and previously learned action-effect combinations which are in contradiction to each other (Kunde, 2001; Koch et al., 2004; Kunde et al., 2004). For example, based on lifelong experience human agents are used to objects that move in the same direction and to the same extent as the body effector controlling these objects. If however, they are confronted with a situation in which these associations are violated, for example by inverting the movements of the controlled object, the anticipated visual effects of the object and proprioceptive effects of the moving body effector are linked to conflicting motor patterns based on one’s learning history resulting in inferior performance (Kunde et al., 2007, 2012; Müsseler and Skottke, 2011; Wirth et al., 2015; Liesner et al., 2020a, b) and downregulation of the proprioceptive effects (Heuer and Rapp, 2012; Liesner and Kunde, 2020). The notion that these action-effect relationships are established over time and through experience suggests that the study of children as still developing agents provides insights into the interdependencies of the mechanisms of ideomotor learning, haptic neglect, and active ownership.

Children have accumulated less experience than adults about motor actions and their effects. Therefore, also the “knowledge” which interoceptive and exteroceptive sensations usually coincide when controlling body-external objects might be developed to a much lesser extent. Consequently, violations of the “common mapping” in cases of interference might also be less likely detected by children. At the same time, children as developing agents should be highly sensitive to current contingencies between their own actions and ensuing perceptual events in the environment, given that they still need to learn these action-effect combinations. In line with this, neuroimaging studies comparing activations in sensorimotor regions between children and adults while performing and observing actions have found lower activation patterns in children than in adults when performing the same tasks suggesting that actions might be represented in a less elaborate way in children than in adults (Mall et al., 2005; Morales et al., 2019).

Children of 3 months show less distinction in terms of event-related potentials between self-produced or externally produced stimuli than is typically observed in adults (Bäß et al., 2008; Baess et al., 2011; Meyer and Hunnius, 2021). Additionally, even children between 7 and 12 years have a strong tendency to report “illusory agency” over events objectively not caused by their actions, a bias that gradually decreases over childhood (e.g., Metcalfe et al., 2010; van Elk et al., 2015). Furthermore, children up to 10 years are unable to integrate multisensory information in an optimal fashion (Ernst and Banks, 2002). Instead, children below this age often display “overintegration” or “overbinding” in which one sensory modality is highly attended and the estimation of the other modality is (almost) completely shifted towards the former one (Gori et al., 2008; Cowie et al., 2016; Nava et al., 2020). All these findings suggest that infants and children up to the age of 10 years do not make the clear distinction between action-contingent and action non-contingent perceptual changes that adults make. Instead, they seem to be biased to ascribe perceptual events to their own actions in a less constrained way than adults. Interestingly, while the active rubber hand illusion occurs in children from 4 years on (Nava et al., 2018), it also seems to be less vulnerable to asynchronous visual and tactile stimulation (Cowie et al., 2013) and sometimes already emerges before stimulation (Nava et al., 2017). These findings fit well with the observed “overintegration” of multisensory information in children of this age and extend these findings to the phenomenon of active ownership. This suggests that the previously discussed limitations for active ownership in adults, especially the one of conflicting interoceptive and exteroceptive information, might be less pronounced in children who still develop a model of typical sensorimotor contingencies. Therefore, children, unlike (young) adults, might integrate interoceptive and exteroceptive signals “unselectively” (i.e., independently of their spatiotemporal matching) and this effect might only gradually become more selective throughout childhood.

Moreover, children between 2.5 and 8 years have considerable problems using tools that move incompatibly to their hands (Contreras-Vidal et al., 2005; Beisert and Daum, 2021), i.e., which create situations with interfering interoceptive sensations from the body effector and exteroceptive (visual) sensations from the tool, which exceed the problems that young adults have. At first glance, this might seem contradictory to the previously discussed findings and the claim that children of this age integrate multisensory information regardless of their (mis)match. However, the claim that interfering sensations are integrated in children and that this interference is seemingly not detected as such does not mean that there would be no interference produced by these sensory inputs at all in children. On the contrary, children might simply not have developed the means to overcome such multisensory conflict. Linking observations of “overintegration” of conflicting external events and performance costs in controlling such events would be a valuable contribution of future research. Additionally, the subject of “haptic neglect” has, to our knowledge, not yet been investigated in children and infants at all which would provide a further interesting testbed for the proposed mechanisms.

## Conclusions and Outlook

In this article, we have reviewed and tried to integrate literature from the fields of the sense of (body) ownership, ideomotor action control, perception and action, and developmental psychology with the aim to provide a description and mechanistic explanations of “active ownership”, i.e., how humans construct a sense of ownership over the effects of their actions. While we reviewed the factors supporting and limiting the feeling of active ownership and possible differences to the factors underlying passive ownership, we suggest that the overlap of interoceptive and exteroceptive sensations is the joint factor shaping the sense of ownership in both cases. Specifically, we argue that conflicting interoceptive and exteroceptive sensations stemming from the same action prevent the experience of active ownership due to compensatory downregulation mechanisms of the system to maintain sufficient motor control. This downregulation is probably less developed in children than in adults. Based on the available developmental studies on the reviewed topics, we suggest that this leads to a relatively unselective integration of interoceptive and exteroceptive sensations in children which are less constrained by the factors we have identified for adults.

While there are various studies providing empirical evidence for the phenomena we have reviewed in isolation, we want to stimulate more integrative research in the fields reviewed in this article to test relationships and commonalities of these phenomena. Specifically, the study of these phenomena in developing agents like children allows us to critically test the predictions made by our approach on how active ownership emerges. While we have so far only looked at children as developing agents more generally, it might be very interesting to compare children of different age groups which should obviously differ in brain maturation (Paus et al., 1999; Gogtay et al., 2004), regarding their experience with sensorimotor contingencies and thus also regarding the effects of interest, as has already been shown in various cross-sectional studies (e.g., Contreras-Vidal et al., 2005; Metcalfe et al., 2010; van Elk et al., 2015). Testing our proposed mechanisms and predictions in different age groups could thus provide the most direct evidence for the relationship between the processes underlying active ownership which we have suggested here.

## Author Contributions

ML: conceptualization, literature search, manuscript—first draft, manuscript—re-writing and editing. N-AH: literature search, manuscript—re-writing and editing. WK: conceptualization, literature search, manuscript—re-writing and editing. All authors contributed to the article and approved the submitted version.

## Conflict of Interest

The authors declare that the research was conducted in the absence of any commercial or financial relationships that could be construed as a potential conflict of interest.

## Footnotes

^1^Regarding the inconsistent use of the term “interoception” in the literature, we want to state here what we refer to by this term: Unlike other authors we do not constrain interoception to the perceptual signals generated only inside the body (e.g., by visceral organs; Craig, 2009; Tsakiris, 2017), but we subsume all perceptual signals under this term which are generated either on or within the biological body like, for example, also tactile signals or proprioception.

## References

[B1] Armel K. C. Ramachandran V. S. (2003). Projecting sensations to external objects: evidence from skin conductance response. Proc. R. Soc. Lond. B Biol. Sci. 270, 1499–1506. 10.1098/rspb.2003.2364 12965016 PMC1691405

[B2] Baess P. Horváth J. Jacobsen T. Schröger E. (2011). Selective suppression of self-initiated sounds in an auditory stream: an ERP study. Psychophysiology 48, 1276–1283. 10.1111/j.1469-8986.2011.01196.x 21449953

[B3] Bahrick L. E. Watson J. S. (1985). Detection of intermodal proprioceptive-visual contingency as a potential basis of self-perception in infancy. Dev. Psychol. 21, 963–973. 10.1037/0012-1649.21.6.963

[B4] Bäß P. Jacobsen T. Schröger E. (2008). Suppression of the auditory N1 event-related potential component with unpredictable self-initiated tones: evidence for internal forward models with dynamic stimulation. Int. J. Psychophysiol. 70, 137–143. 10.1016/j.ijpsycho.2008.06.005 18627782

[B5] Beisert M. Daum M. M. (2021). Compatibility effects in young children’s tool use: learning and transfer. Child Dev. 92, e76–e90. 10.1111/cdev.13455 32864749

[B6] Belin P. Zatorre R. J. Lafaille P. Ahad P. Pike B. (2000). Voice-selective areas in human auditory cortex. Nature 403, 309–312. 10.1038/35002078 10659849

[B7] Bigelow A. E. (1981). The correspondence between self- and image movement as a cue to self-recognition for young children. J. Genet. Psychol. 139, 11–26. 10.1080/00221325.1981.10533432 7288415

[B8] Botvinick M. Cohen J. (1998). Rubber hands ‘feel’ touch that eyes see. Nature 391:756. 10.1038/35784 9486643

[B9] Braun N. Debener S. Spychala N. Bongartz E. Sörös P. Müller H. H.. (2018). The senses of agency and ownership: a review. Front. Psychol. 9:535. 10.3389/fpsyg.2018.00535 29713301 PMC5911504

[B10] Brown H. Adams R. A. Parees I. Edwards M. Friston K. (2013). Active inference, sensory attenuation and illusions. Cogn. Process. 14, 411–427. 10.1007/s10339-013-0571-3 23744445 PMC3824582

[B11] Brownell C. A. Nichols S. R. Svetlova M. Zerwas S. Ramani G. (2010). The head bone’s connected to the neck bone: when do toddlers represent their own body topography? Child Dev. 81, 797–810. 10.1111/j.1467-8624.2010.01434.x 20573105 PMC2892807

[B12] Caharel S. Poiroux S. Bernard C. Thibaut F. Lalonde R. Rebai M. (2002). ERPs associated with familiarity and degree of familiarity during face recognition. Int. J. Neurosci. 112, 1499–1512. 10.1080/00207450290158368 12652901

[B13] Cardinali L. Zanini A. Yanofsky R. Roy A. C. de Vignemont F. Culham J. C.. (2021). The toolish hand illusion: embodiment of a tool based on similarity with the hand. Sci. Rep. 11:2024. 10.1038/s41598-021-81706-6 33479395 PMC7820319

[B14] Cole J. Paillard J. (1995). “Living without touch and peripheral information about body position and movement: studies with deafferented subjects,” in The Body and the Self, eds Bermúdez J. L. Marcel A. J. Eilan N. (Cambridge, MA: The MIT Press), 245–266.

[B15] Contreras-Vidal J. L. Bo J. Boudreau J. P. Clark J. E. (2005). Development of visuomotor representations for hand movement in young children. Exp. Brain Res. 162, 155–164. 10.1007/s00221-004-2123-7 15586275

[B16] Cowie D. Makin T. R. Bremner A. J. (2013). Children’s responses to the rubber-hand illusion reveal dissociable pathways in body representation. Psychol. Sci. 24, 762–769. 10.1177/0956797612462902 23538915

[B17] Cowie D. Sterling S. Bremner A. J. (2016). The development of multisensory body representation and awareness continues to 10 years of age: evidence from the rubber hand illusion. J. Exp. Child Psychol. 142, 230–238. 10.1016/j.jecp.2015.10.003 26601752

[B18] Craig A. D. (2009). How do you feel—now? The anterior insula and human awareness. Nat. Rev. Neurosci. 10, 59–70. 10.1038/nrn2555 19096369

[B19] Critchley H. D. Wiens S. Rotshtein P. Öhman A. Dolan R. J. (2004). Neural systems supporting interoceptive awareness. Nat. Neurosci. 7, 189–195. 10.1038/nn1176 14730305

[B20] Decety J. Lamm C. (2007). The role of the right temporoparietal junction in social interaction: how low-level computational processes contribute to meta-cognition. Neuroscientist 13, 580–593. 10.1177/1073858407304654 17911216

[B21] Desantis A. Weiss C. Schütz-Bosbach S. Waszak F. (2012). Believing and perceiving: authorship belief modulates sensory attenuation. PLoS One 7:e37959. 10.1371/journal.pone.0037959 22666424 PMC3362539

[B22] Dummer T. Picot-Annand A. Neal T. Moore C. (2009). Movement and the rubber hand illusion. Perception 38, 271–280. 10.1068/p5921 19400435

[B23] Ebert J. P. Wegner D. M. (2010). Time warp: authorship shapes the perceived timing of actions and events. Conscious. Cogn. 19, 481–489. 10.1016/j.concog.2009.10.002 19896868 PMC2836403

[B24] Ehrsson H. H. Spence C. Passingham R. E. (2004). That’s my hand! Activity in premotor cortex reflects feeling of ownership of a limb. Science 305, 875–877. 10.1126/science.1097011 15232072

[B25] Elsner B. Hommel B. (2001). Effect anticipation and action control. J. Exp. Psychol. Hum. Percept. Perform. 27, 229–240. 10.1037//0096-1523.27.1.229 11248937

[B26] Ernst M. O. Banks M. S. (2002). Humans integrate visual and haptic information in a statistically optimal fashion. Nature 415, 429–433. 10.1038/415429a 11807554

[B27] Evans N. Blanke O. (2013). Shared electrophysiology mechanisms of body ownership and motor imagery. NeuroImage 64, 216–228. 10.1016/j.neuroimage.2012.09.027 22995776

[B29] Filippetti M. L. Johnson M. H. Lloyd-Fox S. Dragovic D. Farroni T. (2013). Body perception in newborns. Curr. Biol. 23, 2413–2416. 10.1016/j.cub.2013.10.017 24268410 PMC3898688

[B30] Filippetti M. L. Lloyd-Fox S. Longo M. R. Farroni T. Johnson M. H. (2015). Neural mechanisms of body awareness in infants. Cereb. Cortex 25, 3779–3787. 10.1093/cercor/bhu261 25404469 PMC4585515

[B28] Filippetti M. L. Tsakiris M. (2018). Just before I recognize myself: the role of featural and multisensory cues leading up to explicit mirror self-recognition. Infancy 23, 577–590. 10.1111/infa.12236 29937697 PMC6001620

[B31] Flynn E. (2008). Investigating children as cultural magnets: do young children transmit redundant information along diffusion chains? Philos. Trans. R. Soc. B Biol. Sci. 363, 3541–3551. 10.1098/rstb.2008.0136 18799417 PMC2607344

[B32] Fourneret P. Jeannerod M. (1998). Limited conscious monitoring of motor performance in normal subjects. Neuropsychologia 36, 1133–1140. 10.1016/s0028-3932(98)00006-2 9842759

[B33] Gallagher S. (2000). Philosophical conceptions of the self: implications for cognitive science. Trends Cogn. Sci. 4, 14–21. 10.1016/s1364-6613(99)01417-5 10637618

[B34] Gallagher S. Cole J. (1995). Body image and body schema in a deafferented subject. J. Mind Behav. 16, 369–389.

[B35] Gogtay N. Giedd J. N. Lusk L. Hayashi K. M. Greenstein D. Vaituzis A. C.. (2004). Dynamic mapping of human cortical development during childhood through early adulthood. Proc. Natl. Acad. Sci. U S A 101, 8174–8179. 10.1073/pnas.0402680101 15148381 PMC419576

[B36] Gori M. Del Viva M. Sandini G. Burr D. C. (2008). Young children do not integrate visual and haptic form information. Curr. Biol. 18, 694–698. 10.1016/j.cub.2008.04.036 18450446

[B37] Grill-Spector K. Malach R. (2004). The human visual cortex. Annu. Rev. Neurosci. 27, 649–677. 10.1146/annurev.neuro.27.070203.144220 15217346

[B38] Guterstam A. Gentile G. Ehrsson H. H. (2013). The invisible hand illusion: multisensory integration leads to the embodiment of a discrete volume of empty space. J. Cogn. Neurosci. 25, 1078–1099. 10.1162/jocn_a_00393 23574539

[B39] Haggard P. (2017). Sense of agency in the human brain. Nat. Rev. Neurosci. 18, 196–207. 10.1038/nrn.2017.14 28251993

[B40] Heinisch C. Dinse H. R. Tegenthoff M. Juckel G. Brüne M. (2011). An rTMS study into self-face recognition using video-morphing technique. Soc. Cogn. Affect. Neurosci. 6, 442–449. 10.1093/scan/nsq062 20587597 PMC3150855

[B41] Heuer H. Rapp K. (2012). Adaptation to novel visuo-motor transformations: further evidence of functional haptic neglect. Exp. Brain Res. 218, 129–140. 10.1007/s00221-012-3013-z 22328066

[B42] Hohwy J. Paton B. (2010). Explaining away the body: experiences of supernaturally caused touch and touch on non-hand objects within the rubber hand illusion. PloS One 5:e9416. 10.1371/journal.pone.0009416 20195378 PMC2827559

[B43] Hommel B. (2013). “Ideomotor action control: on the perceptual grounding of voluntary actions and agents,” in Action science: Foundations of an Emerging Discipline, eds Prinz W. Beisert M. Herwig A. (Cambridge, MA: MIT Press), 113–136.

[B44] Hughes G. Desantis A. Waszak F. (2013). Mechanisms of intentional binding and sensory attenuation: the role of temporal prediction, temporal control, identity prediction, and motor prediction. Psychol. Bull. 139, 133–151. 10.1037/a0028566 22612280

[B45] Ionta S. Ferretti A. Merla A. Tartaro A. Romani G. L. (2010). Step-by-step: the effects of physical practice on the neural correlates of locomotion imagery revealed by fMRI. Hum. Brain Mapp. 31, 694–702. 10.1002/hbm.20898 19862697 PMC6871054

[B46] James W. (1981). The Principles of Psychology. Cambridge, CA: Harvard University Press.

[B47] Jeannerod M. (2003). The mechanism of self-recognition in humans. Behav. Brain Res. 142, 1–15. 10.1016/s0166-4328(02)00384-4 12798261

[B48] Kaiser J. Schütz-Bosbach S. (2018). Sensory attenuation of self-produced signals does not rely on self-specific motor predictions. Eur. J. Neurosci. 47, 1303–1310. 10.1111/ejn.13931 29738617

[B49] Kalckert A. Ehrsson H. H. (2012). Moving a rubber hand that feels like your own: a dissociation of ownership and agency. Front. Hum. Neurosci. 6:40. 10.3389/fnhum.2012.00040 22435056 PMC3303087

[B51] Kalckert A. Bico I. Fong J. X. (2019a). Illusions with hands, but not with Balloons—Comparing ownership and referral of touch for a corporal and noncorporal object after visuotactile stimulation. Perception 48, 447–455. 10.1177/0301006619839286 30939992

[B52] Kalckert A. Perera A. T.-M. Ganesan Y. Tan E. (2019b). Rubber hands in space: the role of distance and relative position in the rubber hand illusion. Exp. Brain Res. 237, 1821–1832. 10.1007/s00221-019-05539-6 31079236 PMC6584242

[B50] Kalckert A. Ehrsson H. H. (2014). The moving rubber hand illusion revisited: comparing movements and visuotactile stimulation to induce illusory ownership. Conscious. Cogn. 26, 117–132. 10.1016/j.concog.2014.02.003 24705182

[B53] Kilteni K. Maselli A. Kording K. P. Slater M. (2015). Over my fake body: body ownership illusions for studying the multisensory basis of own-body perception. Front. Hum. Neurosci. 9:141. 10.3389/fnhum.2015.00141 25852524 PMC4371812

[B54] Kirsch W. Kunde W. (2019). Impact of action planning on visual and body perception in a virtual grasping task. Exp. Brain Res. 237, 2431–2445. 10.1007/s00221-019-05601-3 31309253

[B55] Kirsch W. Kunde W. Herbort O. (2019). Intentional binding is unrelated to action intention. J. Exp. Psychol. Hum. Percept. Perform. 45, 378–385. 10.1037/xhp0000612 30570318

[B56] Kirsch W. Pfister R. Kunde W. (2016). Spatial action-effect binding. Attent. Percept. Psychophys. 78, 133–142. 10.3758/s13414-015-0997-z 26486641

[B57] Klaffehn A. L. Baess P. Kunde W. Pfister R. (2019). Sensory attenuation prevails when controlling for temporal predictability of self-and externally generated tones. Neuropsychologia 132:107145. 10.1016/j.neuropsychologia.2019.107145 31319119

[B58] Knoblich G. Kircher T. T. J. (2004). Deceiving oneself about being in control: conscious detection of changes in visuomotor coupling. J. Exp. Psychol. Hum. Percept. Perform. 30, 657–666. 10.1037/0096-1523.30.4.657 15301616

[B59] Koch I. Keller P. Prinz W. (2004). The ideomotor approach to action control: implications for skilled performance. Int. J. Sport Exerc. Psychol. 2, 362–375. 10.1080/1612197x.2004.9671751

[B60] Kokkinara E. Slater M. (2014). Measuring the effects through time of the influence of visuomotor and visuotactile synchronous stimulation on a virtual body ownership illusion. Perception 43, 43–58. 10.1068/p7545 24689131

[B61] Kunde W. (2001). Response-effect compatibility in manual choice reaction tasks. J. Exp. Psychol. Hum. Percept. Perform. 27, 387–394. 10.1037//0096-1523.27.2.387 11318054

[B62] Kunde W. Koch I. Hoffmann J. (2004). Anticipated action effects affect the selection, initiation, and execution of actions. Q. J. Exp. Psychol. A 57, 87–106. 10.1080/02724980343000143 14681005

[B63] Kunde W. Müsseler J. Heuer H. (2007). Spatial compatibility effects with tool use. Hum. Factors 49, 661–670. 10.1518/001872007X215737 17702217

[B64] Kunde W. Pfister R. Janczyk M. (2012). The locus of tool-transformation costs. J. Exp. Psychol. Hum. Percept. Perform. 38, 703–714. 10.1037/a0026315 22082214

[B65] Lajoie Y. Paillard J. Teasdale N. Bard C. Fleury M. Forget R.. (1992). Mirror drawing in a deafferented patient and normal subjects: visuoproprioceptive conflict. Neurology 42, 1104–1104. 10.1212/wnl.42.5.1104 1579235

[B66] Lenggenhager B. Pazzaglia M. Scivoletto G. Molinari M. Aglioti S. M. (2012). The sense of the body in individuals with spinal cord injury. PLoS One 7:e50757. 10.1371/journal.pone.0050757 23209824 PMC3510173

[B67] Liepelt R. Dolk T. Hommel B. (2017). Self-perception beyond the body: the role of past agency. Psychol. Res. 81, 549–559. 10.1007/s00426-016-0766-1 27056203

[B68] Liesner M. Kunde W. (2020). Suppression of mutually incompatible proprioceptive and visual action effects in tool use. PLoS One 15:e0242327. 10.1371/journal.pone.0242327 33206706 PMC7673520

[B69] Liesner M. Kirsch W. Kunde W. (2020a). The interplay of predictive and postdictive components of experienced selfhood. Conscious. Cogn. 77:102850. 10.1016/j.concog.2019.102850 31731032

[B70] Liesner M. Kirsch W. Pfister R. Kunde W. (2020b). Spatial action-effect binding depends on type of action-effect transformation. Attent. Percept. Psychophys. 82, 2531–2543. 10.3758/s13414-020-02013-2 32130655 PMC7343754

[B71] Lloyd D. M. (2007). Spatial limits on referred touch to an alien limb may reflect boundaries of visuo-tactile peripersonal space surrounding the hand. Brain Cogn. 64, 104–109. 10.1016/j.bandc.2006.09.013 17118503

[B72] Lucci G. Pazzaglia M. (2015). Towards multiple interactions of inner and outer sensations in corporeal awareness. Front. Hum. Neurosci. 9:163. 10.3389/fnhum.2015.00163 25883564 PMC4381648

[B73] Ma K. Hommel B. (2013). The virtual-hand illusion: effects of impact and threat on perceived ownership and affective resonance. Front. Psychol. 4:604. 10.3389/fpsyg.2013.00604 24046762 PMC3764400

[B74] Ma K. Hommel B. (2015a). Body-ownership for actively operated non-corporeal objects. Conscious. Cogn. 36, 75–86. 10.1016/j.concog.2015.06.003 26094223

[B75] Ma K. Hommel B. (2015b). The role of agency for perceived ownership in the virtual hand illusion. Conscious. Cogn. 36, 277–288. 10.1016/j.concog.2015.07.008 26196450

[B76] Maister L. Hodossy L. Tsakiris M. Shinskey J. L. (2020). Self or (M)other? Infants’ sensitivity to bodily overlap with their mother reflects their dyadic coordination. Child Dev. 91, 1631–1649. 10.1111/cdev.13361 32237153 PMC8651012

[B77] Makin T. R. Holmes N. P. Ehrsson H. H. (2008). On the other hand: dummy hands and peripersonal space. Behav. Brain Res. 191, 1–10. 10.1016/j.bbr.2008.02.041 18423906

[B78] Mall V. Linder M. Herpers M. Schelle A. Mendez-Mendez J. Korinthenberg R.. (2005). Recruitment of the sensorimotor cortex—a developmental FMRI study. Neuropediatrics 36, 373–379. 10.1055/s-2005-873077 16429377

[B79] Maravita A. Spence C. Kennett S. Driver J. (2002). Tool-use changes multimodal spatial interactions between vision and touch in normal humans. Cognition 83, B25–B34. 10.1016/s0010-0277(02)00003-3 11869727

[B80] Maselli A. Slater M. (2013). The building blocks of the full body ownership illusion. Front. Hum. Neurosci. 7:83. 10.3389/fnhum.2013.00083 23519597 PMC3604638

[B81] Memelink J. Hommel B. (2013). Intentional weighting: a basic principle in cognitive control. Psychol. Res. 77, 249–259. 10.1007/s00426-012-0435-y 22526717 PMC3627030

[B82] Merleau-Ponty M. (1982). “The child’s relations with others,” in The Primacy of Perception, ed Edie J. M. (Evanston, IL: Northwestern University Press), 96–155.

[B83] Metcalfe J. Eich T. S. Castel A. D. (2010). Metacognition of agency across the lifespan. Cognition 116, 267–282. 10.1016/j.cognition.2010.05.009 20570251

[B84] Meyer M. Hunnius S. (2021). Neural processing of self-produced and externally generated events in 3-month-old infants. J. Exp. Child Psychol. 204:105039. 10.1016/j.jecp.2020.105039 33341016

[B85] Millière R. Carhart-Harris R. L. Roseman L. Trautwein F. M. Berkovich-Ohana A. (2018). Psychedelics, meditation, and self-consciousness. Front. Psychol. 9:1475. 10.3389/fpsyg.2018.01475 30245648 PMC6137697

[B86] Mitchell R. W. (1993). Mental models of mirror-self-recognition: two theories. New Ideas Psychol. 11, 295–325. 10.1016/0732-118x(93)90002-u

[B87] Mocke V. Weller L. Frings C. Rothermund K. Kunde W. (2020). Task relevance determines binding of effect features in action planning. Attent. Percept. Psychophys. 82, 3811–3831. 10.3758/s13414-020-02123-x 32914340 PMC7593314

[B88] Morales S. Bowman L. C. Velnoskey K. R. Fox N. A. Redcay E. (2019). An fMRI study of action observation and action execution in childhood. Dev. Cogn. Neurosci. 37:100655. 10.1016/j.dcn.2019.100655 31102960 PMC6570413

[B89] Morgan R. Rochat P. (1997). Intermodal calibration of the body in early infancy. Ecol. Psychol. 9, 1–23. 10.1207/s15326969eco0901_1

[B90] Moseley G. L. Olthof N. Venema A. Don S. Wijers M. Gallace A.. (2008). Psychologically induced cooling of a specific body part caused by the illusory ownership of an artificial counterpart. Proc. Natl. Acad. Sci. U S A 105, 13169–13173. 10.1073/pnas.0803768105 18725630 PMC2529116

[B91] Müsseler J. Skottke E.-M. (2011). Compatibility relationships with simple lever tools. Hum. Factors 53, 383–390. 10.1177/0018720811408599 21901935

[B92] Müsseler J. Sutter C. (2009). Perceiving one’s own movements when using a tool. Conscious. Cogn. 18, 359–365. 10.1016/j.concog.2009.02.004 19289291

[B93] Nava E. Bolognini N. Turati C. (2017). The development of a cross-modal sense of body ownership. Psychol. Sci. 28, 330–337. 10.1177/0956797616682464 28080303

[B94] Nava E. Föcker J. Gori M. (2020). Children can optimally integrate multisensory information after a short action-like mini game training. Dev. Sci. 23:e12840. 10.1111/desc.12840 31021495

[B95] Nava E. Gamberini C. Berardis A. Bolognini N. (2018). Action shapes the sense of body ownership across human development. Front. Psychol. 9:2507. 10.3389/fpsyg.2018.02507 30618937 PMC6304390

[B96] O’Kane S. H. Ehrsson H. H. (2021). The contribution of stimulating multiple body parts simultaneously to the illusion of owning an entire artificial body. PLoS One 16:e0233243. 10.1371/journal.pone.0233243 33493178 PMC7833142

[B97] Orne M. T. (1962). On the social psychology of the psychological experiment: with particular reference to demand characteristics and their implications. Am. Psychol. 17, 776–783. 10.1037/h0043424

[B98] Overney L. S. Blanke O. (2009). Impaired imagery for upper limbs. Brain Topogr. 22, 27–43. 10.1007/s10548-008-0065-2 18800243

[B99] Paulus M. (2012). Action mirroring and action understanding: an ideomotor and attentional account. Psychol. Res. 76, 760–767. 10.1007/s00426-011-0385-9 22057651

[B100] Paulus M. (2014). The ideomotor approach to imitative learning (IMAIL) in infancy: challenges and future perspectives. Eur. J. Dev. Psychol. 11, 662–673. 10.1007/s00426-011-0385-9 22057651

[B101] Paulus M. Hunnius S. Van Elk M. Bekkering H. (2012). How learning to shake a rattle affects 8-month-old infants’ perception of the rattle’s sound: electrophysiological evidence for action-effect binding in infancy. Dev. Cogn. Neurosci. 2, 90–96. 10.1016/j.dcn.2011.05.006 22682730 PMC6987660

[B102] Paulus M. van Dam W. Hunnius S. Lindemann O. Bekkering H. (2011). Action-effect binding by observational learning. Psychon. Bull. Rev. 18, 1022–1028. 10.3758/s13423-011-0136-3 21779944 PMC3179589

[B103] Paus T. Zijdenbos A. Worsley K. Collins D. L. Blumenthal J. Giedd J. N.. (1999). Structural maturation of neural pathways in children and adolescents: *in vivo* study. Science 283, 1908–1911. 10.1126/science.283.5409.1908 10082463

[B104] Pazzaglia M. Haggard P. Scivoletto G. Molinari M. Lenggenhager B. (2016). Pain and somatic sensation are transiently normalized by illusory body ownership in a patient with spinal cord injury. Restor. Neurol. Neurosci. 34, 603–613. 10.3233/RNN-150611 27080071

[B105] Perruchoud D. Michels L. Piccirelli M. Gassert R. Ionta S. (2016). Differential neural encoding of sensorimotor and visual body representations. Sci. Rep. 6:37259. 10.1038/srep37259 27883017 PMC5121642

[B106] Pfister R. (2019). Effect-based action control with body-related effects: implications for empirical approaches to ideomotor action control. Psychol. Rev. 126, 153–161. 10.1037/rev0000140 30604990

[B107] Pfister R. Klaffehn A. L. Kalckert A. Kunde W. Dignath D. (2020). How to lose a hand: sensory updating drives disembodiment. Psychon. Bull. Rev. [Epub ahead of print]. 10.3758/s13423-020-01854-0 33300113 PMC8219564

[B108] Pozeg P. Palluel E. Ronchi R. Solcà M. Al-Khodairy A. W. Jordan X.. (2017). Virtual reality improves embodiment and neuropathic pain caused by spinal cord injury. Neurology 89, 1894–1903. 10.1212/WNL.0000000000004585 28986411 PMC5664293

[B109] Renault A. G. Auvray M. Parseihian G. Miall R. C. Cole J. Sarlegna F. R. (2018). Does proprioception influence human spatial cognition? A study on individuals with massive deafferentation. Front. Psychol. 9:1322. 10.3389/fpsyg.2018.01322 30131736 PMC6090482

[B110] Riemer M. Kleinböhl D. Hölzl R. Trojan J. (2013). Action and perception in the rubber hand illusion. Exp. Brain Res. 229, 383–393. 10.1007/s00221-012-3374-3 23307154

[B111] Rohde M. Di Luca M. Ernst M. O. (2011). The rubber hand illusion: feeling of ownership and proprioceptive drift do not go hand in hand. PLoS One 6:e21659. 10.1371/journal.pone.0021659 21738756 PMC3125296

[B112] Romani G. L. Williamson S. J. Kaufman L. (1982). Tonotopic organization of the human auditory cortex. Science 216, 1339–1340. 10.1126/science.7079770 7079770

[B113] Samad M. Chung A. J. Shams L. (2015). Perception of body ownership is driven by Bayesian sensory inference. PLoS One 10:e0117178. 10.1371/journal.pone.0117178 25658822 PMC4320053

[B114] Sanchez-Vives M. V. Spanlang B. Frisoli A. Bergamasco M. Slater M. (2010). Virtual hand illusion induced by visuomotor correlations. PLoS One 5:e10381. 10.1371/journal.pone.0010381 20454463 PMC2861624

[B115] Scandola M. Aglioti S. M. Lazzeri G. Avesani R. Ionta S. Moro V. (2020). Visuo-motor and interoceptive influences on peripersonal space representation following spinal cord injury. Sci. Rep. 10:5162. 10.1038/s41598-020-62080-1 32198431 PMC7083926

[B116] Scandola M. Dodoni L. Lazzeri G. Arcangeli C. A. Avesani R. Moro V.. (2019). Neurocognitive benefits of physiotherapy for spinal cord injury. J. Neurotrauma 36, 2028–2035. 10.1089/neu.2018.6123 30526335

[B117] Shin Y. K. Proctor R. W. Capaldi E. J. (2010). A review of contemporary ideomotor theory. Psychol. Bull. 136, 943–974. 10.1037/a0020541 20822210

[B118] Slater M. Spanlang B. Sanchez-Vives M. V. Blanke O. (2010). First person experience of body transfer in virtual reality. PLoS One 5:e10564. 10.1371/journal.pone.0010564 20485681 PMC2868878

[B119] Sülzenbrück S. Heuer H. (2009). Functional independence of explicit and implicit motor adjustments. Conscious. Cogn. 18, 145–159. 10.1016/j.concog.2008.12.001 19136279

[B120] Suzuki K. Garfinkel S. N. Critchley H. D. Seth A. K. (2013). Multisensory integration across exteroceptive and interoceptive domains modulates self-experience in the rubber-hand illusion. Neuropsychologia 51, 2909–2917. 10.1016/j.neuropsychologia.2013.08.014 23993906

[B122] Tajadura-Jiménez A. Longo M. R. Coleman R. Tsakiris M. (2012). The person in the mirror: using the enfacement illusion to investigate the experiential structure of self-identification. Conscious. Cogn. 21, 1725–1738. 10.1016/j.concog.2012.10.004 23123685 PMC3759963

[B121] Tajadura-Jiménez A. Tsakiris M. (2014). Balancing the “inner” and the “outer” self: interoceptive sensitivity modulates self-other boundaries. J. Exp. Psychol. Gen. 143, 736–744. 10.1037/a0033171 23750913 PMC3848898

[B123] Taub E. (1976). Movement in nonhuman primates deprived of somatosensory feedback. Exerc. Sport Sci. Rev. 4, 335–374. 10.1249/00003677-197600040-00012 828579

[B124] Tong F. Nakayama K. (1999). Robust representations for faces: evidence from visual search. J. Exp. Psychol. Hum. Percept. Perform. 25, 1016–1035. 10.1037//0096-1523.25.4.1016 10464943

[B125] Tsakiris M. (2010). My body in the brain: a neurocognitive model of body-ownership. Neuropsychologia 48, 703–712. 10.1016/j.neuropsychologia.2009.09.034 19819247

[B126] Tsakiris M. (2017). The multisensory basis of the self: from body to identity to others. Q. J. Exp. Psychol. 70, 597–609. 10.1080/17470218.2016.1181768 27100132 PMC5214748

[B128] Tsakiris M. Carpenter L. James D. Fotopoulou A. (2010). Hands only illusion: multisensory integration elicits sense of ownership for body parts but not for non-corporeal objects. Exp. Brain Res. 204, 343–352. 10.1007/s00221-009-2039-3 19820918

[B129] Tsakiris M. Costantini M. Haggard P. (2008). The role of the right temporo-parietal junction in maintaining a coherent sense of one’s body. Neuropsychologia 46, 3014–3018. 10.1016/j.neuropsychologia.2008.06.004 18601939

[B127] Tsakiris M. Haggard P. (2005). The rubber hand illusion revisited: visuotactile integration and self-attribution. J. Exp. Psychol. Hum. Percept. Perform. 31, 80–91. 10.1037/0096-1523.31.1.80 15709864

[B130] Tsakiris M. Prabhu G. Haggard P. (2006). Having a body versus moving your body: how agency structures body-ownership. Conscious. Cogn. 15, 423–432. 10.1016/j.concog.2005.09.004 16343947

[B131] Tsakiris M. Schütz-Bosbach S. Gallagher S. (2007). On agency and body-ownership: phenomenological and neurocognitive reflections. Conscious. Cogn. 16, 645–660. 10.1016/j.concog.2007.05.012 17616469

[B132] van Elk M. Rutjens B. T. van der Pligt J. (2015). The development of the illusion of control and sense of agency in 7-to-12-year old children and adults. Cognition 145, 1–12. 10.1016/j.cognition.2015.08.004 26298422

[B133] van Gorp S. Kessels A. G. Joosten E. A. Van Kleef M. Patijn J. (2015). Pain prevalence and its determinants after spinal cord injury: a systematic review. Eur. J. Pain 19, 5–14. 10.1002/ejp.522 24824334

[B134] van Stralen H. E. van Zandvoort M. J. Hoppenbrouwers S. S. Vissers L. M. Kappelle L. J. Dijkerman H. C. (2014). Affective touch modulates the rubber hand illusion. Cognition 131, 147–158. 10.1016/j.cognition.2013.11.020 24487106

[B135] Verschoor S. A. Hommel B. (2017). Self-by-doing: the role of action for self-acquisition. Soc. Cogn. 35, 127–145. 10.1521/soco.2017.35.2.127

[B136] Voss M. Ingram J. N. Wolpert D. M. Haggard P. (2008). Mere expectation to move causes attenuation of sensory signals. PLoS One 3:e2866. 10.1371/journal.pone.0002866 18682736 PMC2478717

[B137] Want S. C. Harris P. L. (2002). How do children ape? Applying concepts from the study of non-human primates to the developmental study of ‘imitation’in children. Dev. Sci. 5, 1–14. 10.1111/1467-7687.00194

[B138] Waszak F. Cardoso-Leite P. Hughes G. (2012). Action effect anticipation: neurophysiological basis and functional consequences. Neurosci. Biobehav. Rev. 36, 943–959. 10.1016/j.neubiorev.2011.11.004 22108008

[B139] Weser V. Finotti G. Costantini M. Proffitt D. R. (2017). Multisensory integration induces body ownership of a handtool, but not any handtool. Conscious. Cogn. 56, 150–164. 10.1016/j.concog.2017.07.002 28720401

[B140] Wirth R. Pfister R. Janczyk M. Kunde W. (2015). Through the portal: effect anticipation in the central bottleneck. Acta Psychol. 160, 141–151. 10.1016/j.actpsy.2015.07.007 26247333

[B141] Zeugin D. Notter M. P. Knebel J. F. Ionta S. (2020). Temporo-parietal contribution to the mental representations of self/other face. Brain Cogn. 143:105600. 10.1016/j.bandc.2020.105600 32707434

[B142] Zmyj N. Jank J. Schütz-Bosbach S. Daum M. M. (2011). Detection of visual-tactile contingency in the first year after birth. Cognition 120, 82–89. 10.1016/j.cognition.2011.03.001 21458785

[B143] Zopf R. Polito V. Moore J. (2018). Revisiting the link between body and agency: visual movement congruency enhances intentional binding but is not body-specific. Sci. Rep. 8:196. 10.1038/s41598-017-18492-7 29317726 PMC5760573

